# Nanocomposite Perfluorosulfonic Acid/Montmorillonite-Na^+^ Polymer Membrane as Gel Electrolyte in Hybrid Supercapacitors

**DOI:** 10.3390/gels10070452

**Published:** 2024-07-10

**Authors:** Borislava Mladenova, Galin Borisov, Mariela Dimitrova, Desislava Budurova, Maya Staneva, Filip Ublekov, Antonia Stoyanova

**Affiliations:** 1 Institute of Electrochemistry and Energy Systems, Bulgarian Academy of Sciences, Acad. G. Bonchev Str., bl. 10, 1113 Sofia, Bulgaria; borislava.mladenova@iees.bas.bg (B.M.); gal.rusev@iees.bas.bg (G.B.); mariela.dimitrova@iees.bas.bg (M.D.); 2 Institute of Polymers, Bulgarian Academy of Sciences, Acad. G. Bonchev Str., bl. 103 A, 1113 Sofia, Bulgaria; dbudurova@gmail.com (D.B.); mstaneva@gmail.com (M.S.); ublekov.philip@gmail.com (F.U.)

**Keywords:** solid-state supercapacitors, gel polymer membrane, montmorillonite, carbon xerogel, manganese oxide, physicochemical and electrochemical characterization

## Abstract

Solid-state supercapacitors with gel electrolytes have emerged as a promising field for various energy storage applications, including electronic devices, electric vehicles, and mobile phones. In this study, nanocomposite gel membranes were fabricated using the solution casting method with perfluorosulfonic acid (PFSA) ionomer dispersion, both with and without the incorporation of 10 wt.% montmorillonite (MMT). MMT, a natural clay known for its high surface area and layered structure, is expected to enhance the properties of supercapacitor systems. Manganese oxide, selected for its pseudocapacitive behavior in a neutral electrolyte, was synthesized via direct co-precipitation. The materials underwent structural and morphological characterization. For electrochemical evaluation, a two-electrode Swagelok cell was employed, featuring a carbon xerogel negative electrode, a manganese dioxide positive electrode, and a PFSA polymer membrane serving as both the electrolyte and separator. The membrane was immersed in a 1 M Na_2_SO_4_ solution before testing. A comprehensive electrochemical analysis of the hybrid cells was conducted and compared with a symmetric carbon/carbon supercapacitor. Cyclic voltammetric curves were recorded, and galvanostatic charge–discharge tests were conducted at various temperatures (20, 40, 60 °C). The hybrid cell with the PFSA/MMT 10 wt.% exhibited the highest specific capacitance and maintained its hybrid profile after prolonged cycling at elevated temperatures, highlighting the potential of the newly developed membrane.

## 1. Introduction

The rising demand for portable and flexible cutting-edge electronics in modern society is significantly boosting the development of cost-effective, lightweight, and sustainable energy storage systems with high power and energy densities. Hybrid supercapacitors have drawn considerable attention in recent years due to their merits of high-power density, superior rate capability, rapid charging/ discharging rate, long cycle life, etc. [1]. The development of flexible supercapacitors is a relatively new direction in this field, which is of interest for a number of applications, including electronic devices, electric vehicles, cellular phones, etc. [2,3,4,5,6].

The electrolyte is a key component of the supercapacitor, essential for transferring and balancing charges between the positive and negative electrodes. The interaction between the electrodes and the electrolyte greatly influences the state of the electrode–electrolyte interface and the internal structure of the active materials [7]. Therefore, the requirements for the electrolyte are many—it must be stable, have a high ionic conductivity, a wide voltage window, etc. Liquid electrolytes are the most widely used. Solid electrolytes provide a wide window of electrochemical stability and are nonflammable and impermeable, but their low ionic conductivity and poor wetting properties limit their application. Different approaches have been investigated to improve the ionic conductivity, such as the use of a ferroelectric ceramic ion conductor (LiTaO_3_) to lighten the space charge layer and provide an additional Li^+^ transport pathway [8] or the implementation of lithium superconducting nanowires reinforced with high-performance polymer electrolytes [9]. Fast and stable transfer of lithium ions between solid-state electrolytes and electrodes is also a challenge. The formation of an elastic, ultrathin, LiF-rich layer that binds the electrolyte and the lithium anode helps maintain dynamic contact, facilitating ion transfer, promoting uniform lithium deposition, and suppressing side reactions [10]. Other alternatives in this direction are polymer electrolytes, which are solid polymer electrolytes and gel electrolytes.

Gel polymer electrolytes (GPEs) bridge liquid and solid polymer electrolytes (SPEs) and serve as both electrolytes and separators [11]. They combine the benefits of liquid and solid electrolytes, consisting of a polymer matrix, electrolyte salts, and plasticizers, which enhance ionic conductivity by reducing crystalline content [12]. Common GPE materials include polyethylene oxide (PEO), polyvinyl alcohol (PVA), polyacrylonitrile (PAN), and poly(vinylidene fluoride) (PVDF) [13]. As these are often derived from non-renewable sources, researchers are developing alternatives like refractory crosslinking composite GPEs with phosphene and P-N synergistic flame retardants (DPM) to enhance Li^+^ conductivity [14]. Additionally, chlorinating electrolytes for sodium-based dual-ion batteries using ethyl methyl carbonate (EMC) improves oxidative stability and compatibility with both cathodes and anodes by forming Cl-containing interface layers [15]. GPEs are also used in supercapacitors [16].

Solid polymer electrolytes exhibit excellent processability, are lightweight, and safe to use. In this case, the electrolyte works as a separator for the positive and negative electrodes, and hence, these electrolytes show high ionic conductivity [17]. Nafion has been extensively studied for the formation of nano-composite membranes with good performance [18,19]. A solid-state symmetric supercapacitor with Nafion electrolytes has been investigated. The relatively high capacitance performances of the device are explained by several factors, including an excellent adhesion realized between electrodes and the Nafion membrane, homogeneous inter-distribution of carbon/Nafion in electrodes, a good contact between electrodes and end-plates, and fast proton transport in smaller carbon pores. The latter phenomenon can be explained by the favorable influence of water molecules around the Nafion binder/electrolyte that are in contact with the carbon pores [20]. The combination of Nafion with conducting polymers has been demonstrated to enhance the stability of PAN nanofiber electrodes. This improvement is attributed to Nafion’s ability to stabilize the radical cations formed during the charging of the conducting polymer material [21,22]. Natural clays, such as montmorillonite (MMT), with multilayer structure and high surface area, are attractive materials for supercapacitors. Natural MMT consists of hydrated sodium–calcium–aluminum–magnesium silicate hydroxides of approximate formula (Na,Ca)_0.33_(Al,Mg)_2_(Si_4_O_10_)(OH)_2_·nH_2_O, where an octahedral layer of AlO_6_ units is sandwiched between two tetrahedral layers of SiO_4_ units. When the MMT clay is activated with acid, it is used as a solid catalyst for various organic syntheses and is found under the name K10 [23,24]. During the acid treatment, the crystal structure of MMT undergoes several changes: the edges of the crystals open and the octahedral cations (Al or Mg) are washed away from the MMT structure, leading to a gradual destruction of the octahedral layers. Thus, the crystal structure of K10 is characterized by partially disrupted octahedral layers located between the tetrahedral silicate layers, resulting in a highly porous substance with an increase in both surface area and pore diameter [25]. Furthermore, due to the isomorphous substitution of cations, a constant net negative surface charge is known to develop in K10 [26]. A novel hybrid composite based on multi-walled carbon nanotube (MWCNT), acid-leached montmorillonite, and manganese dioxide (MnO_2_) was prepared using 1.0 M tetraethylammonium tetrafluroborate (Et_4_NBF_4_) in acetonitrile (AN) as the electrolyte. The asymmetric supercapacitor showed a high energy density of 171 Wh kg^−1^ at a power density of ~1.98 kW kg^−1^. Such high EDLC performance could possibly be linked to the acid–base interaction of montmorillonite through its surface hydroxyl groups with the tetraethylammonium cation [(C_2_H_5_)4N^+^ or TEA^+^] of the ionic liquid electrolyte. This study demonstrates the excellent potential of clay-based composites for supercapacitor applications [27].

These studies provoked our interest in preparing a composite membrane based on perfluorosulfonic acid (PFSA) and natural MMT. It is well-known that natural MMT is rich in sodium cations. The sodium cationic charges on the surface and interlayers of clay platelets could lead to the promotion of Na^+^-conducting pathways. To maximize this effect, a composite PFSA membrane with maximum well-dispersed MMT particles is needed. The addition of MMT particles could effectively improve the stability of the system interface and interfacial stability between polymer electrolytes and electrodes [28]. A study was conducted on PFSA membranes, both with and without montmorillonite (MMT), to assess their effectiveness as separators in an asymmetric supercapacitor. This supercapacitor featured a manganese dioxide (MnO_2_) positive electrode and a carbon xerogel negative electrode.

## 2. Results and Discussion

### 2.1. Physicochemical Characterization of the MnO_2_ and the Polymer Membrane

**Figure 1:**
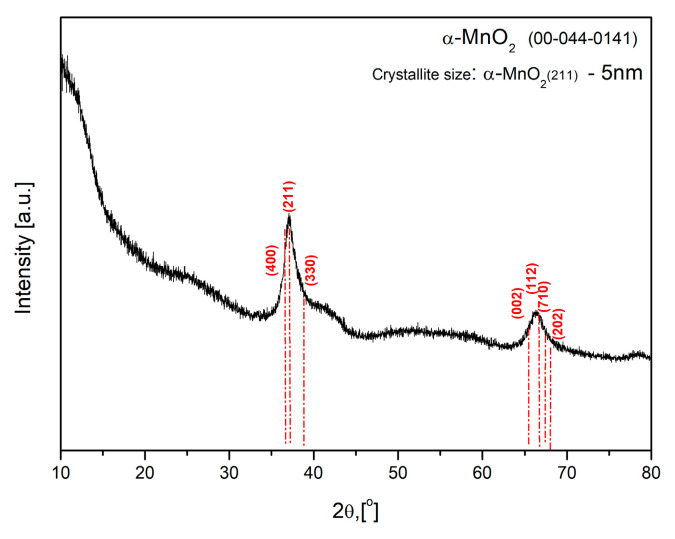
XRD image for α-MnO_2_ nanoparticles.

The X-ray structural characterization of the synthesized MnO_2_ is presented in Figure 1. The XRD diffractogram reveals a low degree of crystallinity and the fine-grained nature of the material established by the manifestation of two broad peaks at 2θ 37.5° and 66.7°, corresponding to (211) and (112) crystallographic planes of MnO_2_ with a structural modification-α (according to ICDD, 2021, No. 00-044-0141 database). These main peaks include other peak positions (at 2θ 36.7°, 39°, 65.1°, 67.6°, and 68.2°) in different directions that did not appear due to the fine dispersion of the α-MnO_2_. In confirmation, the size of the crystallites (t) oriented along the plane (211) is 5 nm, determined by applying Scherer’s formula (t = λ/Bcosθ, where λ (in Å) − 1.54056 is X-ray wavelength; θ (in degrees) is the diffraction angle; B (in rad) is line broadening at the full width at half maximum (FWHM).

**Figure 2:**
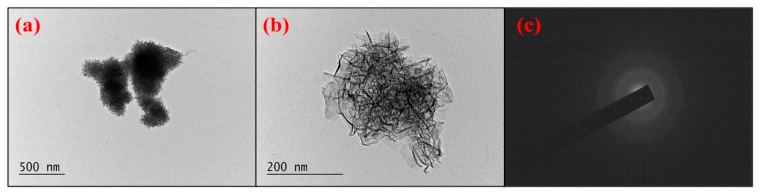
TEM image for α-MnO_2_ nanoparticles at various magnifications (**a**) 10 k, (**b**) 40 k and (**c**) SAED.

The transmission electron microscopy (TEM) image (Figure 2) illustrates the presence of nanosized manganese dioxide particles characterized by varying morphologies such as spherical and rod-shaped configurations. These particles exhibit interconnection, forming a multilayered network structure. The SAED images depict distinct concentric rings, reflecting interplanar spacing within the crystal lattice of α-MnO_2_.

**Figure 3:**
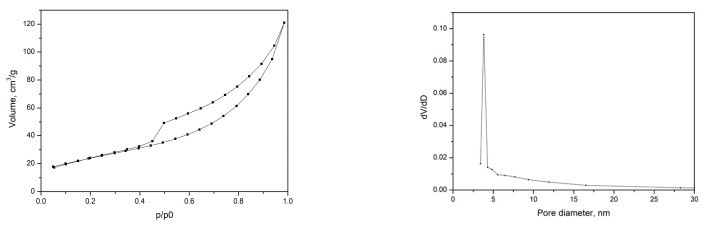
Adsorption–desorption isotherm (**left**) and pore distribution curve of α-MnO_2_ (**right**).

The specific surface area of the synthesized manganese dioxide is 86 m^2^g^−1^. Its average pore diameter is 8.7 nm, a value based on the assumption that the pores have a cylindrical geometry, with *p*/*p*0 = 0.99. MnO_2_ exhibits a narrow pore size distribution in the range between 3 and 18 nm. The adsorption–desorption isotherm (Figure 3) are II and III types, characteristic of macroporous materials according to the classification of IUPAC [29]. The presence of mesopores is also observed.

**Figure 4:**
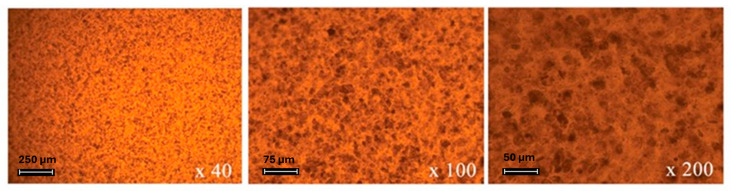
POM images of PFSA/MMT 10 wt.% nanocomposite membrane.

Polarized optical microscopy (POM) studies clearly indicated a homogeneous nanocomposite membrane, displaying water insolubility and well-dispersed MMT particles. The POM images of PFSA/MMT 10 wt.% are presented in Figure 4.

The obtained results show the optimum loading of MMT in the polymer matrix of PFSA is 10 wt.%. PFSA membranes with higher loading led to agglomeration of MMT particles and worsened physicochemical properties.

**Figure 5:**
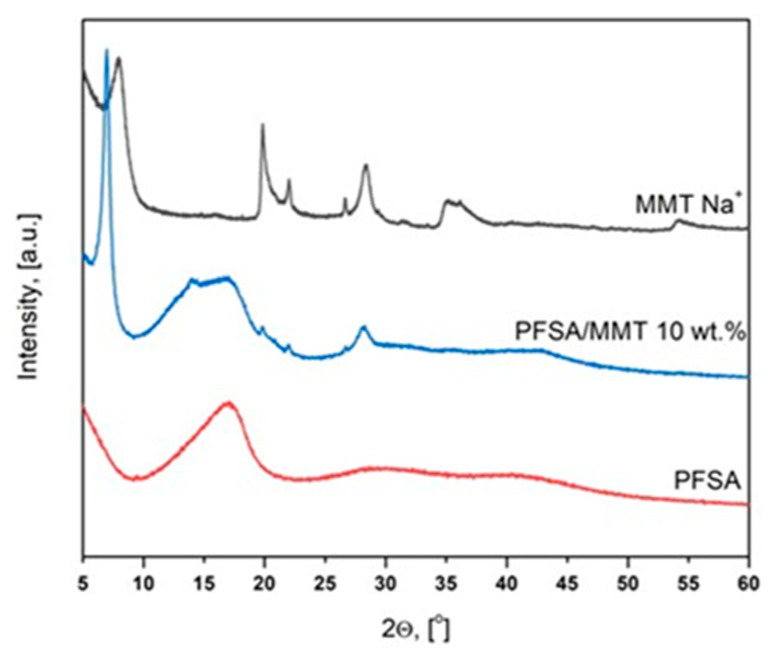
WAXD patterns of PFSA and PFSA/MMT 10 wt.% membranes and MMT.

XRD was employed to identify the intercalated structure of the PFSA/MMT composite membrane. The intercalation of the polymer chains increases the interlayer spacing. It is widely accepted that the driving force for polymer chain intercalation into layered silicate from solution is the entropy gained by the desorption of solvent molecules, which offsets the decreased entropy of the confined, intercalated chains [30]. Figure 5 shows the diffraction patterns of PFSA, MMT Na^+^ and PFSA/MMT 10 wt.%.

The X-ray diffractogram of the synthesized nanocomposite (PFSA/MMT 10 wt.%) reveals the structural transformations that occurred within the clay matrix (MMT Na^+^) after intercalation of the polymeric PFSA chains. These are evident through the appearance of the amorphous halo of the PFSA alongside the peaks of the montmorillonite matrix. Further evidence of the polymer chains’ interposition into the interlayer space of the MMT is a shift in the position of its main peak from 7.97° to 6.96° 2θ. This displacement is due to the increase in the interplanar spacing (d_001_) from 11.08 Å to 12.69 Å, calculated according to Bragg’s law: 2dsinθ = nλ (d_hkl_—interplanar spacing, hkl—Miller indices; θ—Bragg angle; n—order of the interference, normally n = 1; λ—wavelength).

**Figure 6:**
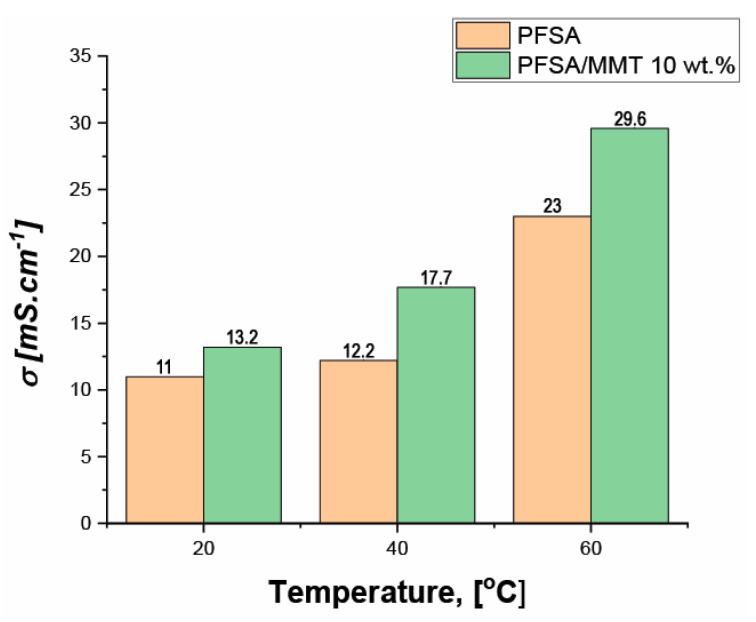
In-plane 4-electrode ion conductivity of neat PFSA and PFSA/MMT 10 wt.% at 20, 40 and 60 °C and 95% RH.

Figure 6 shows the neat PFSA and composite PFSA/MMT 10 wt.% membrane’s ion conductivities at different temperatures and 95% RH. Both membranes exhibit a marked increase in conductivity with increasing temperature. This is because temperature plays a major role in the kinetics of sodium ion motion in the polymeric membrane, the MMT galleries, and the mobility of polymer chains.

In comparison to neat PFSA, the conductivity of PFSA/MMT 10 wt.% is higher at any temperature. At room temperature the ion conductivity of both membranes is similar, but with the temperature increasing, the difference becomes bigger in favor of the composite one. The neat PFSA membrane gives an ion conductivity of 23 ms·cm^−1^ at 60 °C. For the composite membrane, the measured ion conductivity is 29.6 ms·cm^−1^ at 60 °C, i.e., 29% increase. This clearly indicates that the presence of natural MMT plays a crucial role in improving ion conductivity. This phenomenon could be attributed to the sodium ions found usually in MMT interlayer galleries [31]. These galleries served as new ion conduction pathways.

### 2.2. Electrochemical Results

Electrochemical tests, including CV curves and galvanostatic charge/discharge studies at 20 °C, were carried out on the assembled asymmetric supercapacitor cells employing the synthesized membrane with and without MMT. Comparative tests were also conducted on symmetrical supercapacitors equipped with AC carbon electrodes.

**Figure 7:**
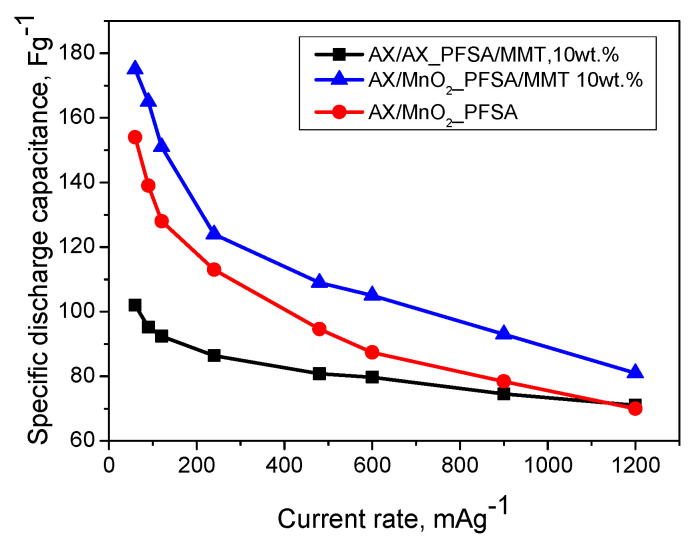
Specific discharge capacitance, calculated from galvanostatic charge/discharge tests, at different current rates for the supercapacitors with PFSA and PFSA/MMT 10 wt.%—membranes at 20 °C.

The results from galvanostatic charge/discharge (GCD) curves at different current rates are shown in Figure 7.

**Figure 8:**
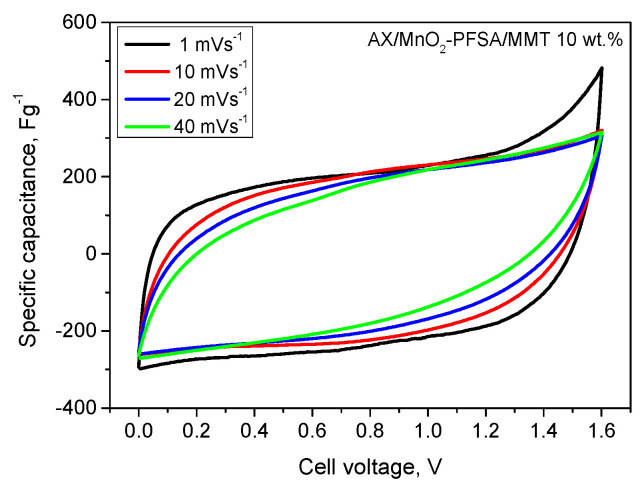
Cyclic voltammetry at different scan rates from 1 to 50 mVs^−1^ for asymmetric supercapacitors and PFSA/MMT 10 wt.%—membrane and 20 °C.

The improvement of the solid-state supercapacitor in an asymmetric configuration (carbon gel in the negative electrode and MnO_2_ in the positive electrode, denoted as AX/MnO_2_) was observed. The mass ratio of the two electrodes was set to 2:1 (AX:MnO_2_), which was found to effectively balance the anodic and cathodic overvoltages [32]. It is evident that the use of the asymmetric configuration of the solid-state supercapacitor presents a superior alternative in terms of electrode composition. The combination of non-faradaic and faradaic redox mechanisms in the case of asymmetric configuration allows for synergistic enhancement of performance. It is also evident that the AX/MnO_2_ asymmetric configuration shows superior performance, with the introduction of MMT into the membrane structure leading to the highest discharge capacitance. The CV profiles of this cell show a typical almost rectangular shape at scan rates from 1 to 40 mVs^−1^ (Figure 8). This suggests the excellent capacitive behavior of this cell [33].

The results obtained illustrate the impact of the intercalated structure of the PFSA/MMT composite membrane. MMT possesses a layered structure, and its integration into the membrane results in an expansion of the distance between the layers and favors improving ion transportation. Moreover, this incorporation enhances both the ionic conductivity and tensile strength of PFSA. The PFSA/MMT composite electrolyte exhibits superior integrated characteristics in terms of ionic conductivity and tensile strength when the mass fraction of MMT is approximately 10 wt.%

**Figure 9:**
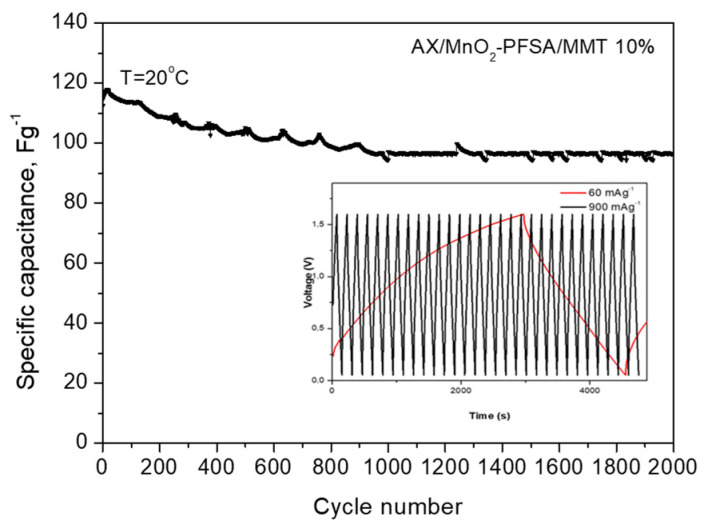
Long—term test of AX/MnO_2_ and PFSA/MMT—membrane supercapacitors at 240 mAg^−1^ and 20 °C, inset figure: voltage profiles at current rates 60 and 900 mAg^−1^.

Cyclic performance emerges as a critical characteristic for assessing SC stability. Discharge cycles were executed at a current density of 240 mAg^−1^. As depicted in Figure 9, the asymmetric cell with a PFSA/MMT electrolyte demonstrates cycling stability with an 85% retention rate for up to 2000 cycles. This result implies that PAMPS/MMT remains stable during charge–discharge cycles and maintains good interfacial contact with MnO_2_ and activated carbon [34]. The voltage profile follows the typical pattern of a hybrid system and also indicates that the developed SC exhibits low resistance. This could be attributed to the well-homogenized MnO_2_/AX composite electrode and the fact that surface reactions in the positive electrode proceed faster and are closer to the electrolyte membrane. Therefore, diffuse species likely travel shorter distances during the charge and discharge phases, aided by the presence of MMT. This observation finds support in the cyclic voltammograms in Figure 8 and the GCD curves in Figure 7. The capacitance of the MnO_2_/AX SC is superior to that of the symmetric SC, although the positive electrode is theoretically less electrically conductive than the carbon electrode, a trend observed in similar solid-state SCs [35].

It is clear that temperature has a significant effect on the electrochemical performance of supercapacitors. Higher temperatures generally result in improved electrochemical performance, including increased capacitance and reduced resistance. Additionally, the results in Figure 6 show that, with increasing temperature, the ionic conductivity of the studied membranes also increases significantly, especially on the composite PFSA/MMT 10 wt.%. This prompted us to perform electrochemical studies at higher temperatures to gather information about the stability of the MMT-embedded membrane and its potential suitability for use at elevated temperatures. Electrochemical tests were performed at 40 °C and 60 °C. After the tests at 60 °C, the tests were repeated at room temperature to assess membrane stability.

**Figure 10:**
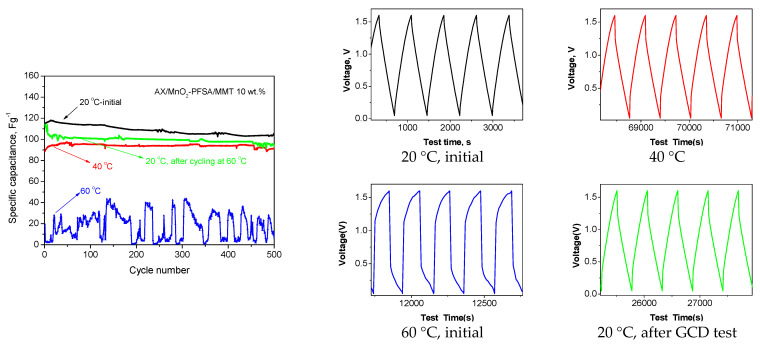
Specific discharge capacitance calculated from galvanostatic charge/discharge tests for AX/MnO_2_—PFSA/MMT 10 wt.%—membrane supercapacitors at 240 mAg^−1^ and voltage profiles at different temperatures.

The results from GCD tests at elevated temperatures are illustrated in Figure 10.

**Figure 11:**
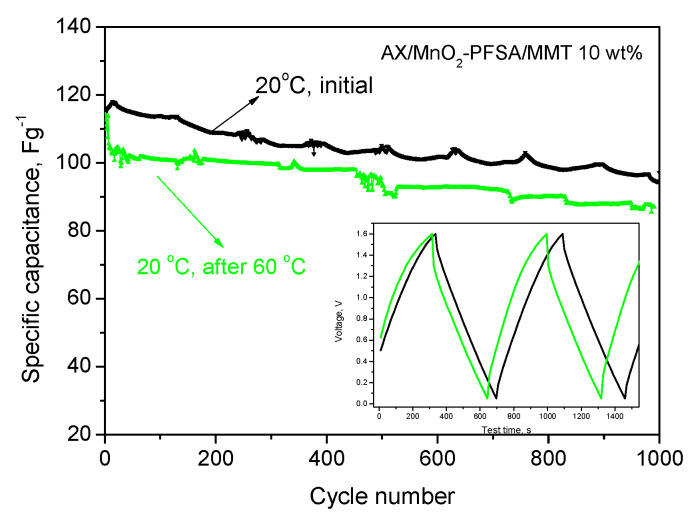
Comparison of discharge capacitance of AX/MnO_2_-PFSA/MMT 10 wt.%—membrane supercapacitor at 240 mAg^−1^ at 20 °C, before and after heating to 60 °C; inset figure: voltage profiles.

The discharge capacitance of the supercapacitor decreases as the temperature rises, with a particularly noticeable decrease at 60 °C, accompanied by significant fluctuations. The voltage profiles depict a significant increase in cell resistance, which naturally impacts its performance. It is worth noting that when the supercapacitor was cooled back to 20 °C from 60 °C, the capacitance almost returned to its original value, with very little reduction. This suggests that certain irreversible processes, such as SO_4_-induced surface redox reactions on the electrodes, occurred during the high-temperature charge–discharge cycles of the supercapacitor [36]. Overall, this reduction is insignificant and underscores the durability of the membrane used, confirming its suitability as an electrolyte and separator in supercapacitor systems. This conclusion is further supported by the conducted long-term test after cooling, as depicted in Figure 11.

One potential reason associated with the observed increase in internal resistance, particularly noticeable at 60 °C, is the generation of gases at elevated temperatures due to electrochemical reactions [37].

## 3. Conclusions

This study investigated nanocomposite gel membranes fabricated with perfluorosulfonic acid (PFSA) ionomer dispersion, both with and without 10 wt.% natural sodium MMT incorporation. Electrochemical evaluation using a two-electrode cell demonstrated improved performance, notably in the hybrid cell with 10 wt.% MMT, which maintained high specific capacitance even after prolonged cycling at elevated temperatures. These results underscore the significant role of natural sodium MMT in enhancing the ion conductivity of the newly developed membrane, likely attributed to the presence of sodium ions in its enlarged interlayer galleries, highlighting its potential for use in energy storage devices.

## 4. Materials and Methods

### 4.1. Preparation of the MnO_2_

Manganese oxide was synthesized by a simple method of chemical precipitation. For the synthesis, KMnO_4_ (Valerus Co., Sofia, Bulgaria) was used as a reducing agent and manganese (II) chloride MnCl_2_ (Valerus Co., Bulgaria) and distilled water were used as a solvent. The mixing of the two solutions of KMnO_4_ and MnCl_2_ results in the formation of a fine precipitate. The obtained suspension is stirred for 1 h at room temperature. During synthesis, two phases precipitate, and the supernatant liquid was washed several times with distilled water in a centrifuge (NEYA-16) for 30 min at 5000 rpm. The precipitate obtained was dried at 80 °C in a vacuum dryer. The starting powder material was subjected to an additional heat treatment for 3 h at 200 °C.

### 4.2. Preparation of Composite PFSA/MMT Membranes

A solution casting method was employed to prepare composite membranes using PFSA (FuMA-Tech, Bietigheim-Bissingen, Germany) and MMT (Cloisites Na^+^, purchased from Southern Clay Products Inc., Louisville, KY, USA), a clay material. The initial solution of PFSA used was 20 wt.% in isopropanol (iPrOH). This solution was diluted to 10 wt.% by adding a solvent while continuously stirring. Subsequently, a specified amount of MMT (10 wt.%) was ultrasonically dispersed into the PFSA solution, followed by mechanical stirring at 60 °C for 2 h to ensure a uniform mixture. The resulting composite mixture was cast onto a glass plate and dried under vacuum at 80 °C for 8 h. After cooling to room temperature, the glass plate was immersed in deionized water to facilitate the peeling of the membrane. Additionally, pure PFSA membranes were fabricated using the same procedure, excluding the addition of clay. The membrane thicknesses were averaged from several measurements taken in different regions of the samples using a Mitutoyo high-accuracy digital micrometer. Before measuring the thickness at room temperature, the membranes were equilibrated in distilled water, and their surfaces were carefully dried with paper. The average membrane thickness is 65 µm ± 2.

### 4.3. Electrode Preparation and Supercapacitor Cell Assembly

The electrodes were prepared in the form of inks and deposited as thin layers by a casting technique. The manganese was used as a positive electrode in the asymmetric cell containing 75 wt.% MnO_2_, 10 wt.% ABG 1005 EG1, 10 wt.% PVDF, 5 wt.% carbon fibers and 1-methyl-2-pyrrolidone solvent. The negative electrode was based on a carbon xerogel (CX) synthesized under laboratory conditions from resorcinol and formaldehyde by microwave synthesis, which has a high surface area (1167 m^2^/g) [38]. The electrodes included 80 wt.% activated carbon (AX), 10 wt.% graphite ABG 1005 EG1, 10 wt.% PVDF and 1-methyl-2-pyrrolidone solvent. The electrodes were subjected to drying at 70 °C and subsequent additional heat treatment for 1 h at 120 °C and 20 min at 160 °C in order to improve their mechanical strength. The mass ratio between the positive and negative electrodes is 1:1. Two types of PFSA membranes, with and without MMT-Na^+^, were used as the electrolyte and separator. Before the electrochemical tests, the membranes were immersed in a 1 M solution of Na_2_SO_4_. For the sake of comparison, symmetrical cells were also assembled with two identical carbon electrodes.

### 4.4. Physicochemical Characterization of Materials

Wide-angle X-ray diffraction (WAXD) scans were obtained using a Bruker D8 Advance ECO diffractometer (Bruker, Billerica, MA, USA), operating at 40 kV and 25 mA in Bragg–Brentano geometry with Ni-filtered Cu Kα radiation and a LynxEye-XE detector over the 2θ range of 5–80°, with a scanning rate of 0.02°·s^−1^. Its morphology was examined by JEOL JEM 2100, an 80–200 kV (Jeol Ltd., Tokyo, Japan) TEM apparatus. The morphology of PFSA/MMT 10 wt.% was visualized and evaluated with a Leica DMLP Optical Microscope and WAXD. A Fumatech MK3 measurement cell equipped with a potentiostat/galvanostat AutoLab model PGSTAT204FRA32M, Metrohm AG, Herisau, Switzerland, was used to perform impedance measurements. The membrane conductivity was determined by four-probe impedance measurements in the frequency range of 0.1–10^−6^ Hz with a 10 mV signal at 20, 40 and 60 °C and 95% relative humidity. Before each experiment, the cell was left for about 1 h to reach thermal equilibrium. The membrane with ~1.5 cm width was placed on top of the four platinum wire electrodes, which were placed on a Teflon disc with a distance of 1 cm between them. The ionic conductivity was calculated according to equation σ = L/R.A, where σ is the ionic conductivity (in mS·cm^−1^), L is the distance between the electrodes (cm), A is the membrane section area (in cm^2^) and R is the impedance of the membrane (in ohms). The porous texture of obtained MnO_2_ was studied using Quantachrome’s AutoSorb iQ apparatus (10215 Timber Ridge Dr, Ashland, VA, USA). The pore size distribution was estimated by using the Barett–Joyner–Halenda method.

### 4.5. Electrochemical Characterization

The electrochemical characteristics of the electrodes were studied in a two-electrode cell (Swagelok-type cell). The capacitor cells were subjected to galvanostatic charge–discharge using an Arbin Instrument System BT-2000 and to long-term cycling with a current of 240 mAg^−1^ at different temperatures (20, 40, 60 °C). Cyclic voltammetry (CV) measurements were also performed with a Multi PalmSens system (model 4, PalmSens BV, Vleugelboot 22, 3991 CL Houten, The Netherlands). The supercapacitor cells were cycled between 0.05 and 1.6 V at a current load increasing stepwise from 60 to 1200 mAg^−1^ for 30 cycles per step. From charge/discharge curves, the specific discharge capacitance (F g^−1^) was calculated following the equation [39]:

C = (I × Δt)/(m × ΔV)(1)

where I (A), Δt (s), m (g) and ΔV (V) are discharge current, discharge time, mass of active material and voltage window, respectively.

## Data Availability

The data that support the findings of this study are available within the articles.

## References

[1] Adil M., Abdelkareem M.A., Sayed E.T., Rodriguez C., Ramadan M., Olabi A.-G. (2022). Progress of Metal Chalcogenides in Supercapacitors. Encycl. Smart Mater..

[2] Feenstra J., Granstrom J., Sodano H. (2008). Energy harvesting through a backpack employing a mechanically amplified piezoelectric stack. MSSP.

[3] Hunang C., Shen C., Tang C., Chang S. (2008). A wearable yarn-based piezo-resistive sensor. Sens. Actuators A Phys..

[4] Wu J., Zhou D., Too C.O., Wallace G.G. (2005). Conducting polymer coated lycra. Synth. Met..

[5] Conway B.E., Pell W. Efficiency Aspects of Charge/Discharge Cycling at Porous Capacitor Electrodes: Behavior of a Hardware Model Circuit. Proceedings of the 8th International Seminar on Double-Layer Capacitors and Similar Energy Storage Devices.

[6] Arbizzani C., Mastragostino M., Scosati B., Nalwa H.S. (1997). Handbook of Organic Conductive Molecules and Polymers.

[7] Holla S., Selvakumar M. (2018). Effect of Different Electrolytes on the Supercapacitor Behavior of Single and Multilayered Electrode Materials Based on Multiwalled Carbon Nanotube/Polyaniline Composite. Macromol. Chem. Phys..

[8] Yuan Y., Chen L., Li Y. (2023). Functional LiTaO_3_ filler with tandem conductivity and ferroelectricity for PVDF-based composite solid-state electrolyte. Energy Mater. Devices.

[9] Peng J., Lu D., Wu S., Yang N., Cui Y., Ma Z., Liu M., Shi Y., Sun Y., Niu J. (2024). Lithium Superionic Conductive Nanofiber-Reinforcing High-Performance Polymer Electrolytes for Solid-State Batteries. J. Am. Chem. Soc..

[10] Yang N., Cui Y., Su H., Peng J., Shi Y., Niu J., Wang F. (2023). A Chemically Bonded Ultraconformal Layer between the Elastic Solid Electrolyte and Lithium Anode for High-performance Lithium Metal Batteries. Angew. Chem..

[11] Zhu M., Wu J., Wang Y., Song M., Long L., Siyal S.H., Yang X., Sui G. (2019). Recent advances in gel polymer electrolyte for high-performance lithium batteries. J. Energy Chem..

[12] Quartarone E., Mustarelli P. (2011). Electrolytes for solid-state lithium rechargeable batteries: Recent advances and perspectives. Chem. Soc. Rev..

[13] Fan L., Wei S., Li S., Li Q., Lu Y. (2018). Recent progress of the solid-state electrolytes for high-energy metal-based batteries. Adv. Energy Mater..

[14] Du Y., Liu X., Chen L., Yin S., Xie Y., Li A., Liang X., Luo Y., Wu F., Mei Y. (2023). 3D hierarchical fireproof gel polymer electrolyte towards high-performance and comprehensive safety lithium-ion batteries. Chem. Eng. J..

[15] Lin Y., Shang J., Liu Y., Wang Z., Bai Z., Ou X., Tang Y. (2024). Chlorination Design for Highly Stable Electrolyte toward High Mass Loading and Long Cycle Life Sodium-Based Dual-Ion Battery. Adv. Mater..

[16] Novakov C., Kalinova R., Veleva S., Ublekov F., Dimitrov I., Stoyanova A. (2023). Flexible Polymer-Ionic Liquid Films for Supercapacitor Applications. Gels.

[17] Lamba P., Singh P., Singh P., Singh P., Bharti, Kumar A., Gupta M., Kumar Y. (2022). Recent advancements in supercapacitors based on different electrode materials: Classifications, synthesis methods and comparative performance. J. Energy Storage.

[18] Navarra M.A., Croce F., Scrosati B. (2007). New, high temperature superacid zirconia-doped Nafion™ composite membranes. J. Mater. Chem..

[19] Allodi V., Brutti S., Giarola M., Sgambetterra M., Navarra M.A., Panero S., Mariotto G. (2016). Structural and Spectroscopic Characterization of A Nanosized Sulfated TiO_2_ Filler and of Nanocomposite Nafion Membranes. Polymers.

[20] Lufrano F., Staiti P. (2004). Performance improvement of Nafion based solid state electrochemical supercapacitor. Electrochim. Acta.

[21] RSong Y., Park J.H., Sivakkumar S.R., Kim S.H., Ko J.M., Park D.Y., Jo S.M., Kim D.Y. (2007). Supercapacitive properties of polyaniline/Nafion/hydrous RuO_2_ composite electrodes. J. Power Sources.

[22] Kim B.C., Ko J.M., Wallace G.G. (2008). A novel capacitor material based on Nafion-doped polypyrrole. J. Power Sources.

[23] Kaur N., Kishore D. (2012). Montmorillonite: An efficient, heterogeneous and green catalyst for organic synthesis. J. Chem. Pharm. Res..

[24] Jin T., Zhang S., Li T. (2002). Transesterification of β-ketoesters with alcohols catalyzed by montmorillonite K-10. Green Chem..

[25] Jha A., Garade A.C., Shirai M., Rode C.V. (2013). Metal cation-exchanged montmorillonite clay as catalysts for hydroxyalkylation reaction. Appl. Clay Sci..

[26] Tombácz E., Szekeres M. (2004). Colloidal behavior of aqueous montmorillonite suspensions: The specific role of pH in the presence of indifferent electrolytes. Appl. Clay Sci..

[27] Maiti S., Pramanik A., Chattopadhyay S., De G., Mahanty S. (2016). Electrochemical energy storage in montmorillonite K10 clay based composite as supercapacitor using ionic liquid electrolyte. J. Colloid Interface Sci..

[28] Kim J.W., Ji K.S., Lee J.P., Park J.W. (2003). Electrochemical characteristics of two types of PEO-based composite electrolyte with functional SiO_2_. J. Power Sources.

[29] Sing K.S.W. (1985). Reporting physisorption data for gas/solid systems with special reference to the determination of surface area and porosity (Recommendations 1984). Pure Appl. Chem..

[30] Ray S.S., Okamoto M. (2003). Polymer/layered silicate nanocomposites: A review from preparation to processing. Prog. Polym. Sci..

[31] Uddin F. (2008). Clays, Nanoclays, and Montmorillonite Minerals. Metall. Mater. Trans. A.

[32] Rey-Raap N., Flores-López S., Santos-Gómez L., Brigandì A., Thomas M., Stoyanova A., Lufrano A., Arenillas A. (2023). Graphene Doped Carbon-Gels and MnO_2_ for Next Generation of Solid-State Asymmetric Supercapacitors. ChemElectroChem.

[33] Rey-Raap N., Angel Menéndez J., Arenillas A. (2014). RF xerogels with tailored porosity over the entire nanoscale. Microporous Mesoporous Mater..

[34] Wang J., Yu X., Wang C., Xiang K., Deng M., Yin H. (2017). PAMPS/MMT composite hydrogel electrolyte for solid-state supercapacitors. J. Alloys Compd..

[35] Thomas M., Veleva S., Karamanova B., Brigandì A., Rey-Raap N., Arenillas A., Stoyanova A., Lufrano F. (2023). Highly stable and reliable asymmetric solid-state supercapacitors with low self-discharge rates. SM&T.

[36] Wang J., Feng S.P., Yang Y., Hau N.Y., Munro M., Ferreira-Yang E., Chen G. (2015). Thermal charging phenomenon in electrical double layer capacitors. Nano Lett..

[37] Conway B.E. (1999). Electrochemical Supercapacitors: Scientific Fundamentals and Technological Applications.

[38] Karamanova B., Mladenova E., Thomas M., Rey-Raap N., Arenillas A., Lufrano F., Stoyanova A. (2023). Electrochemical Performance of Symmetric Solid-State Supercapacitors Based on Carbon Xerogel Electrodes and Solid Polymer Electrolytes. Gels.

[39] Wang T., Zhang S., Yan X., Lyu M., Wang L., Bell J. (2017). and and Wang, H.; 2-Methylimidazole-Derived Ni–Co Layered Double Hydroxide Nanosheets as High Rate Capability and High Energy Density Storage Material in Hybrid Supercapacitors. ACS Appl. Mater. Interfaces.

